# Antibiotic Resistance Patterns of Bacterial Isolates from Blood in San Francisco County, California, 1996-1999

**DOI:** 10.3201/eid0802.010102

**Published:** 2002-02

**Authors:** Susan S. Huang, Brian J. Labus, Michael C. Samuel, Dairian T. Wan, Arthur L. Reingold

**Affiliations:** * University of California San Francisco, San Francisco, California, USA; † California Department of Health Services, San Francisco, California, USA; ‡ University of California Berkeley, Berkeley, California, USA

**Keywords:** antibiotic resistance, MRSA, VRE, bacteremia, county surveillance

## Abstract

Countywide antibiotic resistance patterns may provide additional information from that obtained from national sampling or individual hospitals. We reviewed susceptibility patterns of selected bacterial strains isolated from blood in San Francisco County from January 1996 to March 1999. We found substantial hospital-to-hospital variability in proportional resistance to antibiotics in multiple organisms. This variability was not correlated with hospital indices such as number of intensive care unit or total beds, annual admissions, or average length of stay. We also found a significant increase in methicillin-resistant *Staphylococcus aureus*, vancomycin-resistant *Enterococcus*, and proportional resistance to multiple antipseudomonal antibiotics. We describe the utility, difficulties, and limitations of countywide surveillance.

Many national sampling and hospital surveillance systems exist to monitor antimicrobial resistance patterns in bacteria (*1*–*4*). Previously, organisms resistant to multiple antibiotics were largely confined to hospital settings and were typically described through studies involving single hospitals or intensive care units (ICUs). These single-hospital studies often reported substantially different resistance patterns from one another (*5*–*7*). National surveillance systems provided key data on large-scale resistance trends, but, similarly, continued to show marked variability in proportional resistance among participating hospitals (*1*–*3*,*8*,*9*).

We hypothesized that countywide surveillance data would not only provide information on changes in bacterial antimicrobial resistance but also potentially identify hospital demographic data to account for interhospital variability in resistance patterns. Additionally, countywide surveillance should provide greater insight into the relationship between single hospitals and their neighboring communities as data accumulate to support the spread of resistant organisms from the hospital into the community and vice versa. Morgan et al. (*8*) reported data from Wales that 66% of patients colonized or infected with methicillin-resistant *Staphylococcus aureus* (MRSA) are being discharged to their homes, leading to risks of intrafamilial transmission (*10*–*12*). Goetz et al. (*6*) showed that health-care workers similarly bring resistant organisms home. Additionally, outpatient dialysis units, rehabilitation centers, and outpatient intravascular devices have been shown to be reservoirs of colonization with MRSA and vancomycin-resistant *Enterococcus* (VRE) in many patients in the community (*13*–*16*).

MRSA and VRE colonization and infection in the absence of hospital risk factors are also being increasingly recognized in the community (*17*–*22*). Day-care centers and isolated communities may play a notable role (*20*,*23*). Patients colonized from these community reservoirs can subsequently cause hospital outbreaks after admission (*24*).

As hospital and community colonization and infection begin to exert pressure on one another, single-hospital surveillance data may become less useful in isolation. Countywide surveillance may provide more insight into the sources and extent of outbreaks and prompt focused investigations into the spread and containment of resistant organisms.

We conducted an observational study to evaluate the changes in antibiotic resistance in selected bacteria isolated from blood in San Francisco County, California, to determine if these changes were associated with specific hospital demographics and to define the utility, limitations, and potential areas of improvement in a county-based surveillance system.

## Methods

All bacterial strains recovered from blood were identified from available microbiology department records of all 13 hospitals in San Francisco County from January 1, 1996, to March 31, 1999. For three hospitals (*4*,*5*,*9*), data from 1996 had been purged and were no longer available. For each isolate, data were collected on organism type and susceptibility pattern. Information was also obtained on the ward, age, and gender of the patient. Only the first positive blood culture of a given species was included for a single patient throughout the study period, regardless of susceptibility pattern. Cultures positive for *S. epidermidis* were considered representative of clinical bacteremia if at least two isolates with identical susceptibility patterns were obtained from a minimum of two separate sets of blood cultures. Each such set was considered a single bacteremic event. All other cultures positive for *S. epidermidis* were excluded, as were other common skin contaminants (e.g., *Propionibacterium acnes, Peptostreptococcus*, *Corynebacterium*).

All but one hospital used automated systems (VITEK [bioMerieux Vitek, Hazelwood, MO] or Microscan [Baxter Laboratories, West Sacramento, CA]) for the susceptibility testing of gram-negative bacteria. All hospitals used Kirby-Bauer disk-diffusion techniques for the evaluation of susceptibility profiles for *Streptococcus pneumoniae* and other streptococcal species according to National Committee for Clinical Laboratory Standards (NCCLS) guidelines. Kirby-Bauer results were supplemented by e-testing. One hospital (*12*) performed all susceptibility testing using Kirby-Bauer disk-diffusion techniques. All microbiology laboratories used MIC breakpoints established by the NCCLS. The annual number of blood culture sets processed by each microbiology laboratory was also obtained.

Data from five hospitals were obtained as text files or Microsoft Excel (Redmond, WA) files and subsequently imported into a Microsoft Access (Redmond, WA) database. Data from four hospitals were obtained in printed form, scanned as image files, converted into text files using TextBridge Pro 98 (ScanSoft Inc., Peabody, MA), and imported into the database. All scanned entries were verified for accuracy. Data from the remaining four hospitals were obtained from stored index cards, entered manually into the database, and verified for accurate entry.

We also obtained bed size and census data for all 13 hospitals in San Francisco County. Total hospital admissions were tabulated from quarterly administrative records from 1996 through 1998. For each hospital, average length of hospital stay was obtained from publicly available resources (*25*).

### Data Analyses

The number of isolates for each species was tabulated for each year and for the entire study period. Only species for which the total number of countywide isolates exceeded 100 during the study period were further evaluated. For each organism, proportional annual resistance to an antibiotic was calculated as the yearly number of organisms with intermediate or full resistance divided by the total number of organisms isolated in San Francisco County that year. Because of laboratory variability in susceptibility testing, not all isolates are included in descriptions of proportional resistance.

Means were calculated from the annual countywide percentages in each of the 4 years studied. Percent annual resistance was determined for any antibiotic tested in ≥50 isolates and analyzed for increasing or decreasing annual trend from 1996 through 1999. Data for 1999 were based on the first quarter culture results. Strains with full or intermediate resistance to an antibiotic were counted as resistant in all statistical analyses.

Organisms demonstrating increasing or decreasing annual resistance to a given antibiotic (p<0.05) were further described by calculating mean proportional resistance over the study period according to categories of hospital, ward, patient age (in 10-year intervals), and patient gender. Spearman rank tests were used to determine any correlation between hospital indices (beds, annual admissions, average length of stay) and proportional antibiotic resistance. P values were not adjusted for the effect of multiple comparisons. P values remained unchanged with respect to alpha level (0.05) after removal of the three hospitals missing data from 1996.

## Results

A total of 11,573 bacterial strains were recovered from blood cultures by the 13 hospitals. After excluding duplicate cultures, we had 8,072 remaining clinical isolates.

Information on hospital size, census, and blood culture volume is provided in Table 1. Despite being distinct and nonadjoining, hospitals 4 and 5 are reported together because of unified microbiology and administrative centers. Hospital 13 is a skilled nursing facility. On average, 74,600 sets of blood cultures were processed each year in San Francisco County; 9.9% of these were positive for bacterial species.

**Table 1:** Hospital size and census, San Francisco County, 1996 to 1999

Hospital	# beds	# ICU^a^ beds	Yearly admissions	Average length of stay (days)	Blood culture sets/year	% + blood cultures
1	482	47	20,340	6.6	16,325	11
2	371	32	14,305	3.8	9,108	9
3	304	30	18,843	7.5	12,929	13
4,5^b^	302	31	10,893	5.8	10,800	6
6	284	12	5,939	8.3	3,580	12
7	253	15	7,982	7.3	4,865	9
8	240	15	9,887	7.6	5,475	6
9	221	18	6,139	6.6	2,582	11
10	209	19	6,655	6.7	4,974	14
11	209	8	3,293	9.3	2,515	10
12	59	7	2,260	5.4	1,464	13
13^c^	1,280	0	1,211	351.2	N/A	

^a^ICU beds includes medical, surgical, cardiac, and neurologic adult critical care.

^b^Administration, microbiology laboratories merged. Average number per hospital given.

^c^Skilled nursing facility.

**Figure 1:**
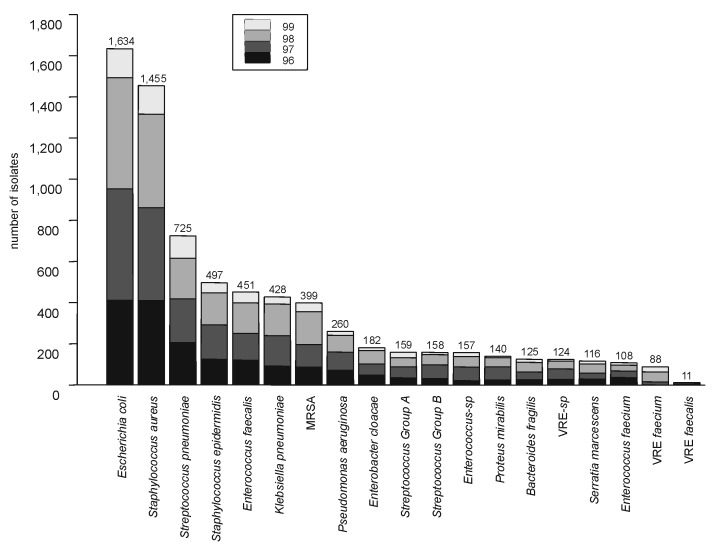
All bacterial species that were isolated from blood in >100 persons, January 1996 through March 1999, all hospitals in San Francisco County, California. Each bar is divided by yearly totals. Total number of isolates obtained during the study period is given above each bar. MRSA = methicillin-resistant *Staphylococcus aureus*; VRE = vancomycin-resistant *Enterococcus.*

Of the 8,072 isolates, *Staphylococcus aureus* (1,858), *Escherichia coli* (1,634), and *S. pneumoniae* (725) were the most common organisms. The numbers of *S. aureus, Enterococcus faecalis, Bacteroides fragilis, E. coli,* and *Serratia marcescens* increased annually during the 4-year period. Fourteen species had >100 isolates and were considered for further analysis (Figure 1). These 14 organisms accounted for 85% to 86% of all yearly totals.

### Gram-Positive Organisms

**Figure 2:**
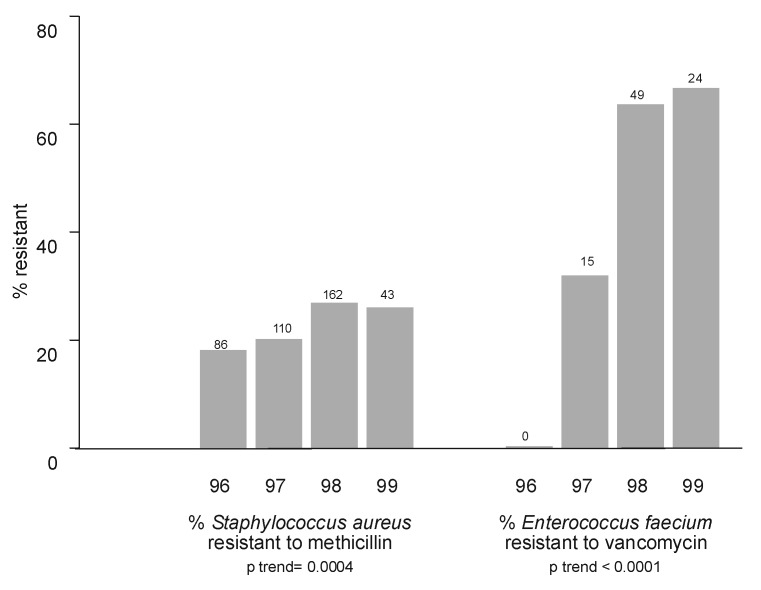
Increase in percentage of *Staphylococcus aureus* isolates resistant to methicillin and increase in percent of *Enterococcus faecium* resistant to vancomycin on a yearly basis from 1996 through the first quarter of 1999. Number of isolates per year, regardless of susceptibility, appears above each bar. Tests of trend showed significant increases in percent resistance for both organisms.

The proportion of MRSA and *E. faecium* resistant to vancomycin (VRE *faecium*) increased annually (Figure 2). Countywide, the proportion of MRSA isolates rose from 18.1% (1996) to 26.1% (1999) (p<0.001). In total, MRSA constituted 22.4% of all *S. aureus* isolates, including 19.6% of emergency department isolates and 15.9% of isolates in the outpatient setting (Table 2). Methicillin resistance in *S. aureus* isolates was <15% in patients <20 years of age and >20% in all other age groups, with the exception of 30- to 39-year-olds (17.6%) (Table 3). Proportional resistance did not vary by patient gender.

**Table 2:** Proportional^a^ resistance of highly resistant organisms by ward,^b^ San Francisco County, 1996 to 1999

Ward	Methicillin- resistant *Staphylococcus aureus*	Vancomycin- resistant *Enterococcus faecium*	Penicillin- resistant *Streptococcus pneumoniae*
Skilled nursing facility	38.0% (100)	66.7% (6)	8.3% (12)
Med/surg ICU	27.2% (298)	50.0% (58)	13.3% (75)
Pediatric ICU	30.3% (33)	25.0% (4)	0% (2)
Med/surg floors	21.8% (709)	47.8% (92)	12.0% (166)
Pediatric floors	9.1% (33)	60.0% (5)	40.0% (10)
Emergency department	19.6% (424)	22.7% (22)	9.5% (305)
Outpatient	15.9% (171)	12.5% (8)	23.6% (72)
Other/unknown	28.6% (7)	100% (1)	32.4% (34)
Total	22.4% (1,782)	44.9% (196)	13.6 (678)

^a^Proportional resistance refers to the proportion of isolates of that species that is resistant to the indicated antibiotic.

^b^Total number of species isolates given in parentheses. Percentages for small numbers of total isolates should be cautiously interpreted.

ICU = intensive care unit.

**Table 3:** Proportional^a^ resistance of highly resistant organisms by age,^b^ San Francisco County, 1996 to 1999

Age group (years)	Methicillin-resistant (%) *Staphylococcus aureus*	Vancomycin-resistant (%) *Enterococcus faecium*	Penicillin- resistant (%) *Streptococcus pneumoniae*
≤10	14.3 (98)	22.2 (9)	19.3 (83)
10-19	8.1 (37)	50.0 (4)	20.0 (5)
20-29	21.7 (83)	50.0 (8)	0 (26)
30-39	17.6 (250)	42.1 (19)	13.4 (142)
40-49	21.8 (353)	41.4 (29)	8.7 (138)
50-59	24.8 (234)	43.8 (32)	11.5 (78)
60-69	29.1 (227)	45.5 (44)	10.2 (49)
70-79	24.8 (258)	55.9 (34)	15.6 (64)
≥80	22.8 (237)	41.2 (17)	17.2 (64)

^a^Proportional resistance refers to the proportion of isolates of that species that is resistant to the indicated antibiotic.

^b^Total number of species isolates given in parentheses. Percentages for small numbers of total isolates should be cautiously interpreted.

During the study period, 124 VRE isolates and 157 vancomycin-sensitive *Enterococcus* isolates were unspeciated. Among the speciated *E. faecium* isolates, the percentage resistant to vancomycin rose from 0% to 66.7% in the 4-year period (p<0.001). VRE *faecium* was most frequently isolated from inpatient adult wards, but six isolates were cultured from emergency department and outpatient settings. Over 66% of *E. faecium* isolates from skilled nursing facilities were resistant to vancomycin (Table 2). VRE isolates exceeded 40% in all age groups with the exception of ≤10-year-olds (22.2%) (Table 3). Of note, VRE *faecium* isolates showed increasing annual resistance to doxycycline (from 30% to 68%; p = 0.02).

**Figure 3:**
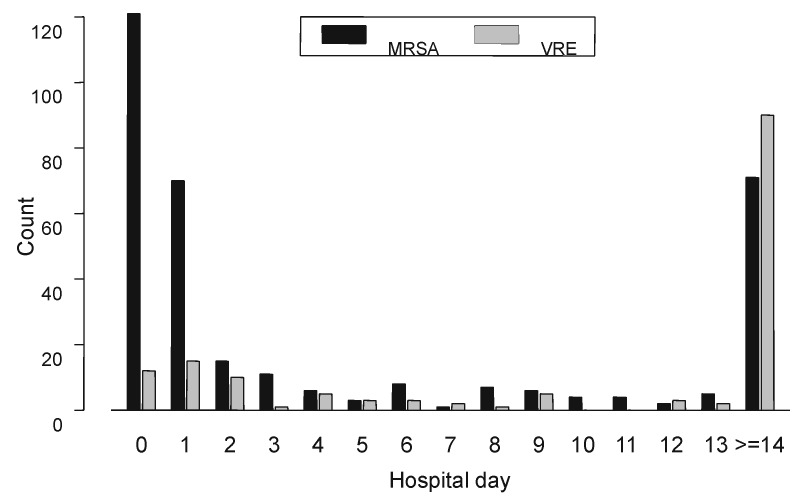
Plot of the number of methicillin-resistant *Staphylococcus aureus* (MRSA) and vancomycin-resistant *Enterococcus* (VRE) isolates by hospital day of admission. An early peak is noted, corresponding to patients entering the hospital with MRSA or VRE bacteremia. Subsequent cases likely represent nosocomial acquisition.

Even after the skilled nursing facility (hospital 13) was excluded, the percentage of MRSA and VRE *faecium* isolates varied substantially among individual hospitals (MRSA 12.5% to 37.5%, VRE *faecium* 12.5% to 80.0%). There was no correlation between proportional resistance and number of ICU or total hospital beds, number of annual admissions, or average length of ICU or total hospital stay. However, for both organisms, there was increasing proportional resistance among adult wards in the following order: outpatient wards, emergency department, medical and surgical floors, medical and surgical ICUs, and skilled nursing facility wards. Notably, a substantial number of VRE and MRSA isolates were cultured within the first 24 to 48 hours of hospital admission (Figure 3).

Of the 678 isolates of *S. pneumoniae*, 13.6% were resistant to penicillin. Proportional resistance increased from 10.8% (21 isolates) in 1996 to 14.6% (27 isolates) in 1998, but this increase was not statistically significant. Proportional resistance was highest at the extremes of age (16.4% in patients >70 years of age and 19.3% in patients <10 years of age (Table 3).

### Gram-Negative Organisms

Countywide, *E. coli* (1,634 isolates), *Klebsiella pneumoniae* (428 isolates), and *Pseudomonas aeruginosa* (260 isolates) were the most frequently isolated gram-negative bacilli. This was true in all inpatient and outpatient wards with the exception of skilled nursing facilities, where *Proteus mirabilis* was the most common gram-negative isolate after *E. coli*. Proportional resistance by ward for selective gram-negative organisms is shown in Table 4.

**Table 4:** Proportional resistance of selected gram-negative organisms by ward^,a^ San Francisco County, 1996 to 1999

	*Escherichia coli*- cefazolin	*E. coli-*ciprofloxacin	*Enterococcus cloacae*-ceftazidime	*Pseudomonas aeruginosa* *-*ciprofloxacin	*P. aeruginosa* *-*ceftazidime	*P. aeruginosa* *-*imipenem	*Serratia marcescens* *-*ceftazidime
Skilled nursing facility	9.1% (88)	2.2% (92)	50% (6)	8.3% (12)	25% (12)	20.0% (10)	40.0% (5)
Med/surg ICU	8.4% (166)	1.9% (160)	53.4% (43)	21.5% (65)	15.9% (63)	11.9% (59)	12.1 (33)
Pediatric ICU	18.5% (27)	0% (28)	18.2% (11)	0% (13)	7.7% (13)	9.1% (11)	0% (11)
Med/surg floors	8.4% (586)	4.3% (564)	47.2% (53)	19.1% (89)	12.5% (88)	12.2% (74)	9.5% (21)
Pediatric floors	25.0% (16)	0% (16)	27.3% (11)	0% (10)	0% (10)	0% (8)	0% (6)
Emergency department	8.4% (536)	2.6% (549)	32.3% (31)	23.5% (34)	5.9% (34)	6.7% (30)	0% (17)
Outpatient	8.6% (117)	1.6% (123)	4.8% (21)	4% (25)	4.0% (25)	0% (22)	0% (15)
Other/unknown	11.1% (18)	0% (18)	0% (0)	0% (1)	0% (1)	0% (1)	0% (0)
Total	8.8% (1,554)	2.9% (1,550)	38.1% (176)	16.5% (249)	11.4% (246)	9.8% (215)	7.3% (109)

^a^Total number of species isolates given in parentheses. Percentages for small numbers of total isolates should be cautiously.

ICU = intensive care unit

Among *E. coli* isolates, resistance to trimethoprim-sulfamethoxazole averaged 28% and resistance to ciprofloxacin averaged 3%. There was no resistance to fluoroquinolones among *E. coli* isolates from pediatric wards. Increasing annual resistance to ticarcillin-clavulanate was seen in both *E. coli* (6% to 16%, p = 0.03) and *K. pneumoniae* (0% to 18%, p = 0.007) isolates.

**Figure 4:**
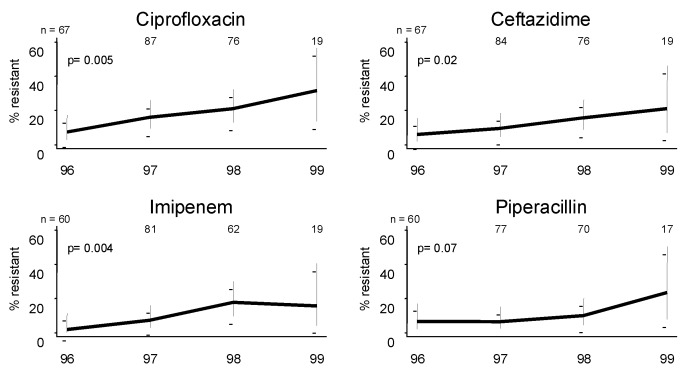
Yearly percent resistance to ciprofloxacin, ceftazidime, imipenem, and piperacillin in *Pseudomonas aeruginosa* isolates from blood. Increasing proportional resistance occurred in three of the four antibiotics commonly used to treat this organism. Annual number of isolates tested to each antibiotic is given at the top of each graph.

*P. aeruginosa* isolates showed increasing annual countywide resistance to ciprofloxacin (7% to 21%, p = 0.005), ceftazidime (6% to 16%, p = 0.02), and imipenem (2% to 18%, p = 0.004) (Figure 4). Resistance to each of these three antibiotics exceeded 10% in adult ICU and adult medical and surgical wards. In fact, in these settings, ciprofloxacin resistance approached 20% countywide. No isolates resistant to ciprofloxacin were cultured from pediatric wards (Table 4). Resistance to gentamicin (15%) and piperacillin-tazobactam (12%) also increased but was not statistically significant.

There were 182 *E. cloacae* and 116 *S. marcescens* isolates from January 1996 through March 1999. Ciprofloxacin resistance averaged 4% among *E. cloacae* isolates and 6% among *S. marcescens* isolates. *S. marcescens* isolates also showed increasing annual proportional resistance to gentamicin (0% to 14%, p = 0.02) and piperacillin (4% to 29%, p = 0.01).

Resistance to ceftazidime, which can be predictive of inducible and extended-spectrum-beta-lactamases, was found in the following overall mean proportions in the study period: *E. coli* (1%), *P. mirabilis* (1%), *K. pneumoniae* (1%), *S. marcescens* (8%), *P. aeruginosa* (13%), and *E. cloacae* (39%). Only *P. aeruginosa* isolates demonstrated an increasing linear annual trend (p = 0.02).

## Discussion

San Francisco County has a population of approximately 735,000 and covers 46.7 square miles (*26*). It comprises multiple racial and ethnic groups (black 10.9%, Hispanic 13.9%, Asian 29.1%, and Native American 0.5%) and is served by 13 hospitals. We have shown that county surveillance of bacterial resistance is a useful addition to local hospital surveillance, particularly as antibiotic resistant bacteria increasingly spread from hospital to hospital and into the community at large.

Across the county, annual proportions of MRSA and VRE isolates significantly increased over the 4-year period. Pseudomonal strains resistant to fluoroquinolones, ceftazidime, or imipenem also increased annually. These data allow us to distinguish countywide outbreaks and trends from single-hospital changes in resistance patterns, and enable infection control efforts to expand or narrow to the appropriate scale. With awareness programs, county surveillance can broaden physicians’ knowledge of their hospital’s effects on the community, as well as the effects of neighboring hospitals on resistance patterns in their particular hospital.

Additionally, county surveillance that includes subcategorization of isolates by ward is invaluable in identifying patients at high risk and locations for transmission of resistant bacteria. Not surprisingly, we report our highest proportion of MRSA and VRE isolates from ICU and nursing home units. Nevertheless, ward variability across hospitals was substantial. Large interhospital differences can lead to further study of ward practices that foster or abate transmission. Awareness can prompt hospital infection control personnel to ensure well-described preventive measures such as swabbing and isolation precautions for VRE and MRSA in ICU settings (*27*–*30*) and nasopharyngeal swabbing and eradication of MRSA in hemodialysis wards (*1*,*31*). We also identify several MRSA and VRE isolates from outpatient and emergency department settings. Whether or not these represent true community-acquired strains or strains from patients recently released from hospital settings, they suggest that highly resistant bacterial outpatient infections and infectivity are increasing, a result consistent with recent studies (*19*,*21*,*22*).

We also evaluated whether the wide variability in the proportions of resistant bacteria among San Francisco hospitals was linked to hospital indices. In contrast to previous nationwide sampling studies, none of this variability was correlated with the number of hospital ICU beds (*3*), total beds (*1*,*2*,*31*), annual admissions (*32*), or annual mean length of stay. This may be due to our small number of hospitals, leading to limited power to detect such correlations. Alternatively, local community and hospital factors (e.g., increasing care of moderately ill patients at home *[8]*, increasing home intravenous antibiotics *[15]*, active transfer of patients between hospitals [*33**,**34*], and community-acquired resistant organisms) may now be diminishing the effect of hospital size, census and length of stay on proportional resistance.

There are many additional advantages to a countywide surveillance system for antimicrobial resistance. First, it can help reassess therapy. The 28% trimethoprim-sulfamethoxazole resistance in *E. coli* raises questions about the optimal empiric treatment of urinary tract infections in patients at high risk for bacteremia or urosepsis. Likewise, distinguishing resistance in the outpatient versus inpatient setting can guide empiric therapy in the appropriate setting.

Second, in studying a larger populace, we can obtain sufficient numbers to study uncommon organisms. Similarly, countywide surveillance provides a means to identify and confirm novel resistant pathogens. In our study, one MRSA isolate with intermediate resistance to vancomycin and three vancomycin-resistant *S. epidermidis* isolates were noted in microbiology laboratory reports. As with the organisms recently reported in the United States (*35*–*37*), these were reported from nontertiary hospitals. Nevertheless, our reports are unconfirmed and likely represent laboratory error. However, if surveillance could be expedited to real-time use, such reports could be investigated and confirmed rather than dismissed. At its worst, this suggests that highly resistant organisms may be escaping deserved attention and reaction.

Third, countywide surveillance engenders further hypotheses and research regarding interhospital and community transmission of resistant organisms. For example, our finding that 20% of emergency department *S. aureus* isolates are methicillin-resistant provides a flag to further study which county areas have the highest percentages of resistant *S. aureus* and which risk factors are involved (e.g., recent hospital admission *[**21**,**22**]*, associated hemodialysis centers [*8**,**38*] or nursing homes *[**21**,**22**]*, or intravenous drug use [*39*]). The finding that *P. mirabilis* bacteremia is more common in nursing home wards raises questions about preventing urinary tract infections in that setting. The relative lack of fluoroquinolone resistance in pediatric gram-negative isolates is likely due to the avoidance of fluoroquinolones in children because of potential detrimental cartilage effects. The study of fluoroquinolone-resistant organisms during the transition years from pediatric to adult medicine may provide insight into the quantity and duration of antibiotic needed to produce selection pressure, as well as the speed and durability of emerging resistance.

Our surveillance method had several limitations. Chart review would have been an invaluable addition in distinguishing between community-acquired and recent hospital or nursing home acquisition of resistant organisms. Second, we did not collect or confirm isolates; thus, although our results reflect microbiologic data actually presented to ordering physicians, they are subject to laboratory differences in speciation and susceptibility determination. Notably, not all organisms were fully speciated or tested against the antibiotics of interest. Fortunately, this occurred in a small proportion of bacteria. Third, we do not provide information on antibiotic use, which is known to be a major determinant of bacterial antibiotic resistance. Fourth, countywide trends can be driven by trends seen in the largest hospitals, particularly since smaller hospitals often lack sufficient numbers of isolates to make statistical analyses meaningful. This was notable for our data on proportional increases in VRE, which was largely driven by three hospitals. On the contrary, MRSA trends were not limited to a few hospitals, nor were they limited to the largest hospitals in the county. No hospital showed a significant decreasing trend in the proportion of MRSA isolates.

Areas for improvement include methods to computerize microbiologic data storage in a universal format. This would expedite surveillance and allow real-time collection and identification of unusually resistant organisms, as well as provide sentinel data regarding countywide outbreaks. In addition, linking of patient information to microbiologic data would have expedited acquisition of sex, gender, and ward information. Furthermore, despite NCCLS guidelines, a fair amount of variability exists in laboratory practices and susceptibility panels. Further standardization of these practices would help ensure the reliability of merging data among hospitals.

Without a doubt, the greatest utility of countywide surveillance lies in its ability to ask screening questions that prompt a more thorough investigation of specific hospitals, wards, or age groups at particular risk for acquiring or transmitting highly resistant organisms.

We have shown how several such questions were raised by our surveillance of bacteremias in San Francisco County and described its many advantages. We have further defined our limitations and difficulties in performing such surveillance, in the hope that this will be helpful to further similar surveillance efforts.
